# Particulate Matter and Incident Chronic Kidney Disease in Japan: The Ibaraki Prefectural Health Study (IPHS)

**DOI:** 10.31662/jmaj.2024-0032

**Published:** 2024-06-03

**Authors:** Kei Nagai, Shin Araki, Toshimi Sairenchi, Kayo Ueda, Kazumasa Yamagishi, Masayuki Shima, Kouhei Yamamoto, Hiroyasu Iso, Fujiko Irie

**Affiliations:** 1 Department of Nephrology, Hitachi General Hospital, Hitachi, Japan; 2 Department of Nephrology, Faculty of Medicine, University of Tsukuba, Tsukuba, Japan; 3 Graduate School of Engineering, Osaka University, Suita, Japan; 4 Medical Science of Nursing, Dokkyo Medical University School of Nursing, Shimotsuga, Japan; 5 Department of Public Health, Institute of Medicine, and Health Services Research and Development Center, University of Tsukuba, Tsukuba, Japan; 6 Department of Hygiene, Graduate School of Medicine, Hokkaido University, Hokkaido, Japan; 7 Department of Public Health, School of Medicine, Hyogo Medical University, Nishinomiya, Japan; 8 Department of Environmental Engineering, Graduate School of Engineering, Kyoto University, Kyoto, Japan; 9 Institute of Global Health Policy Research (iGHP), National Center for Global Health and Medicine, Tokyo, Japan; 10 Tsuchiura Public Health Center of Ibaraki Prefectural Government, Tsuchiura, Japan

**Keywords:** chronic kidney disease, particulate matter, estimated glomerular filtration rate

## Abstract

**Introduction:**

Global health hazards caused by air pollution, such as chronic kidney disease (CKD), have been gaining attention; however, air pollution-associated CKD has not been explored in Japan.

**Methods:**

We examined 77,770 men and women with estimated glomerular filtration rate (eGFR) ≥60 ml/min/1.73 m^2^ in the Ibaraki Prefecture who participated in annual community-based health checkups from 1993 at 40-75 years old and were followed up through December 2020. The outcome was newly developed kidney dysfunction with eGFR of <60 ml/min/1.73 m^2^ during follow-up. To assess air pollution, a PM_2.5_ exposure model was employed to estimate yearly means at 1 × 1-km resolution, converted into means at the municipal level. Hazard modeling was employed to examine PM_2.5_ concentrations in residential areas as a risk factor for outcomes.

**Results:**

Participants were distributed across 23 municipalities in the Ibaraki Prefecture, with PM_2.5_ concentrations between 16.2 and 33.4 μg/m^3^ (mean, 22.7 μg/m^3^) in 1987-1995 as the exposure period. There were 942 newly developed kidney dysfunctions during follow-up. Based on 1987-1995 PM_2.5_ concentrations as the baseline exposure, the multivariate-adjusted hazard ratio per 10-μg/m^3^ increase in PM_2.5_ for newly developed kidney dysfunction was 1.02 (95%CI, 0.80-1.24) in men and 1.19 (95%CI, 0.95-1.44) in women.

**Conclusions:**

Elevated PM_2.5_ did not represent a significant risk factor for incident CKD in a prefecture in Japan.

## Introduction

Epidemiological studies on cardiovascular disease and ambient air concentrations of particulate matter with a diameter of ≤2.5 μm (PM_2.5_), predominantly originating from combustion sources, have been carried out by numerous researchers since the 1990s and have provided a solid base of evidence ^(1), (2), (3), (4)^. Considering the pathophysiology of cardio-renal syndrome, the involvement of PM_2.5_ in chronic kidney disease (CKD) has recently been attracting attention ^(5), (6), (7)^. Considering the links between air pollution and kidneys, experimental evidence has shown that exhaust particles might cause oxidative stress, endothelial dysfunction, and immune inflammation such as the production of tumor necrosis factor α, which leads to endothelial damage; in turn, progressive and cumulative kidney damage and an increased long-term risk of adenine-induced CKD mice model ^(8)^. However, epidemiological studies on associations between exposure to PM_2.5_ and the risk of CKD remain scant in contrast to studies on cardiovascular disease ^(9), (10), (11), (12)^. Recently published results from systematic reviews and meta-analyses have indicated PM_2.5_ as a risk factor for CKD ^(13), (14)^. The publications selected to evaluate the risk of CKD based on PM_2.5_ concentration were limited to those from the United States, Korea, and Taiwan, and studies on links between PM_2.5_ and CKD in Japan remain lacking. Since concentrations of PM_2.5_ vary widely between different countries, different municipalities within a country, and even different areas within a municipality ^(5)^, epidemiological studies on a country-by-country or region-by-region basis are thus important. This study therefore examined whether an association exists in Japan between PM_2.5_ and the development of CKD from a longitudinal perspective study.

## Materials and Methods

### Study design and study population

This prospective observational cohort study is being carried out as part of the Ibaraki Prefectural Health Study (IPHS) ^(15), (16)^. Ibaraki Prefecture is located adjacent to the Tokyo Metropolitan Area; it covers 6,097 km^2^ (representing 1.6% of Japan’s total land area), and it has a population of approximately 28 million people. The southern part of the prefecture is urban and within 1 hour’s commuting distance of Tokyo, and the central and northern parts are rural and predominantly agricultural. Hence, the mean annual PM_2.5_ concentration in the prefecture is high in the south and low in the north. Thirty-eight of the 85 municipalities that existed in the prefecture in 1993 were selected as target areas. Informed consent to conduct an epidemiological study based on guidelines of the Council for International Organizations of Medical Science was obtained from community representatives. We included 97,047 residents (33,133 men and 63,914 women) in Ibaraki Prefecture who participated in annual community-based health checkups beginning in 1993 at 40-75 years old. These health checkups were performed by local municipalities based on Japan’s Health Service Law for the Aged. After excluding 2,254 cases with incomplete data and 17,023 cases with an estimated glomerular filtration rate (eGFR) of <60 ml/min/1.73 m^2^ at baseline, the number of final subjects was 77,770 (28,405 men and 49,365 women). This study was conducted according to the guidelines of the Declaration of Helsinki, and the original study protocol was approved by the ethics committee at Ibaraki Prefectural Office (approval no. R3-4) and then approved by the University of Tsukuba (#1628-1).

### Exposure assessment

For this study, PM_2.5_ concentration in 1993 was applied for the background exposure index. To assess air pollution, a national-scale PM_2.5_ exposure model was utilized to estimate monthly means at a 1 × 1-km resolution across Japan for the period from 1987 to 2016 ^(17)^. Briefly, a neural network model was developed using various predictors against the monitored PM_2.5_ concentrations. The estimates were evaluated to be accurate with *R*^2^ values greater than 0.73 through various validation approaches. Gridded PM_2.5_ concentrations were averaged across the municipal level in each fiscal year. Individuals were assigned the average PM_2.5_ exposure from 1987 to 1995 in the municipality of their residence in 1993.

### Follow-up and outcomes

The participants were followed annually from the baseline in 1993 until the development of kidney dysfunction or the end of 2020. Maximum and median durations of follow-up were thus 27.7 and 22.5 years. Over the study period, the proportion of subjects lost to follow-up was 4.6%. Details regarding the methods applied for mortality surveillance have been reported previously ^(15), (16)^. The outcome was newly developed kidney dysfunction with eGFR of <60 ml/min/1.73 m^2^ during follow-up. Serum creatinine was measured by the modified method of Jaffe’s reaction using the automated analyzer (RX-30, Nihon Denshi Inc., Tokyo, Japan). The eGFR was calculated by using the abbreviated equation developed at Cleveland Clinic laboratory for the Modification of Diet in Renal Disease Study as follows: GFR (ml/min/1.73 m^2^) = 186.3 × age^−0.203^ × serum creatinine level^−1.154^ × (0.742 if female) ^(18)^.

### Potential confounders

Potential confounding factors were basically selected according to our previous research analyzing cardiovascular deaths in IPHS ^(16)^. However, the cohort does not have any information on social contexts that may influence the development of CKD. For example, other cohort studies have analyzed social factors, including educational levels, income, marital status, education, and occupation as covariates ^(9), (10), (11)^. In this study, the selected factors in this study were age, sex, hypertension category ^(19)^, antihypertensive treatment, cigarette smoking (never, past, occasional, and habitual smoker), abnormal glucose tolerance (hyperglycemia and/or diabetes treatment), alcohol intake (never, occasional, and habitual drink), body mass index, serum total cholesterol, high-density lipoprotein cholesterol, use of lipid-lowering drugs, and dipstick proteinuria in the baseline year. As background, the mean values and prevalence of these potential confounding factors were calculated (Table 1).

**Table 1.** Study Population.

Particulate matter concentration (mean, range, μg/m^3^)		First quartile 19.9 (19.6-20.0)	Second quartile 20.3 (20.1-20.8)	Third Quartile 22.2 (21.1-25.0)	Fourth Quartile 28.9 (26.5-31.8)
Men	
Population at risk	Persons	5,350	7,307	6,053	9,695
Age	years	60 ± 10	60 ± 10	60 ± 10	60 ± 10
Body mass index	kg/m^2^	23.3 ± 3.0	23.2 ± 2.9	23.2 ± 2.9	23.2 ± 3.0
Systolic blood pressure	mmHg	135 ± 18	135 ± 18	136 ± 17	137 ± 17
Diastolic blood pressure	mmHg	79 ± 11	80 ± 11	81 ± 11	82 ± 11
Use of antihypertensive drugs	%	19.0	18.1	19.4	19.6
Use of hypoglycemic drugs	%	5.7	3.6	3.7	3.7
Total cholesterol	mg/dl	193 ± 35	193 ± 35	190 ± 35	193 ± 35
High-density lipoprotein	mg/dl	54.1 ± 15.5	54.1 ± 15.5	54.1 ± 15.5	54.1 ± 15.5
Triglyceride	mg/dl	142 ± 97	151 ± 97	151 ± 97	151 ± 97
Use of lipid-lowering drugs	%	0.9	1.3	1.4	1.1
Current smoking	%	49.1	50.6	51.4	53.6
Daily alcohol drink	%	50.7	50.1	53.6	57.5
Women				
Population at risk	Persons	8,689	11,806	11,030	17,840
Age	Years	56 ± 10	56 ± 10	56 ± 10	56 ± 10
Body mass index	kg/m^2^	23.5 ± 3.1	23.6 ± 3.2	23.5 ± 3.2	23.4 ± 3.2
Systolic blood pressure	mmHg	130 ± 18	130 ± 18	131 ± 18	131 ± 18
Diastolic blood pressure	mmHg	77 ± 11	77 ± 10	78 ± 11	78 ± 11
Use of antihypertensive drugs	%	16.1	16.3	15.9	16.6
Use of hypoglycemic drugs	%	1.7	2.0	2.0	1.7
Total cholesterol	mg/dl	205 ± 35	209 ± 35	205 ± 35	205 ± 35
High-density lipoprotein	mg/dl	58.0 ± 15.5	58.0 ± 15.5	58.0 ± 15.5	58.0 ± 15.5
Triglyceride	mg/dl	133 ± 71	133 ± 80	133 ± 80	124 ± 80
Use of lipid-lowering drugs	%	2.9	3.0	2.7	2.9
Current smoking	%	3.8	4.7	5.9	5.1
Daily alcohol drink	%	3.4	2.9	4.0	4.2

### Statistical analysis

Hazard ratios (HRs) and 95% confidence intervals (95% CIs) of baseline difference in PM_2.5_ concentration as a continuous value (per 10-μg/m^3^ increase) for the outcomes were calculated with adjustment for age and other potential confounders using Cox proportional hazards modeling. As there were sex differences in previous studies regarding the risk of PM_2.5_ for cardiovascular mortality in IPHS ^(16)^, a sex stratification was also applied in the present analysis of the development of CKD. Because PM_2.5_ concentrations declined over the course study period, we preliminary carried out a sensitivity analysis by PM_2.5_ exposure in a single year from 1987 to 1995 of the municipal (data not shown), and exposure level of any single year showed no obvious differences in trends for CKD incident risk; then, we decided to assign the average PM_2.5_ exposure level from 1987 to 1995. All statistical analyses were carried out using SAS version 9.4 (SAS Institute, Cary, NC, USA).

## Results

**Figure 1.**
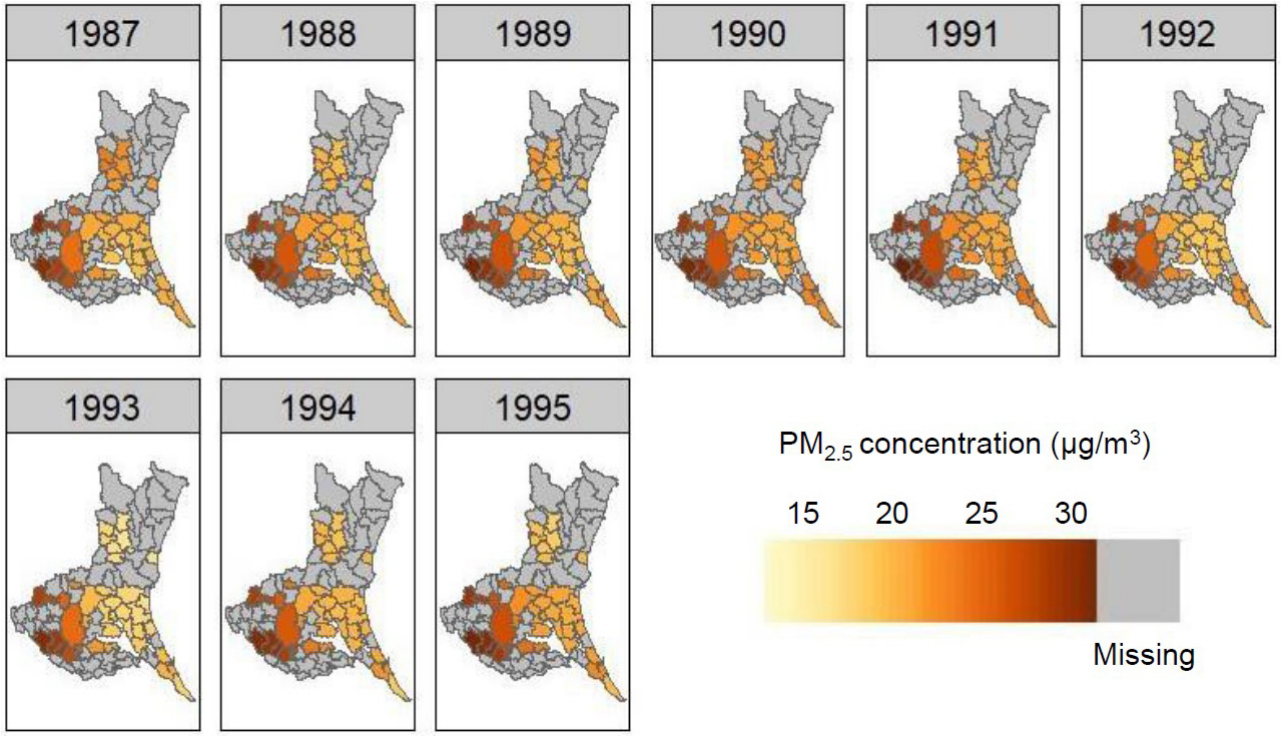
Ambient PM_2.5_ concentration in the study region We previously developed a national-scale PM_2.5_ exposure model for Japan using measurements recorded between 2014 and 2016 to estimate means at a resolution of 1 × 1 km for the years 1987-2015. Data were reconstructed for suitability for the Ibaraki Prefectural Health Study, presenting a heat map for 1987-1995 as the exposure period. The original analytical methods and general results are described elsewhere ^(17)^.

**Figure 2.**
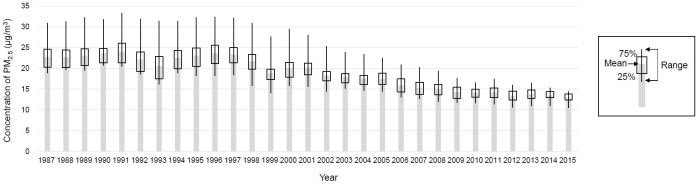
Trends in PM2.5 concentrations in the cohort study area. We developed a national-scale PM2.5 exposure model for Japan using measurements recorded between 2014 and 2016 to estimate means at 1 × 1-km resolution for 1987 through 2015. The data were reconstructed to be suitable for The Ibaraki Prefectural Health. The original analytical methods and general results were described in the reference ^(17)^.

Table 1 lists the baseline characteristics of study subjects categorized by sex (men, 37%) and PM_2.5_ concentration. Figure 1 shows the distribution of PM_2.5_ during 1987-1995 in the 23 municipalities where study subjects resided in Ibaraki Prefecture, with PM_2.5_ concentrations varying from 18.9 to 31.0 μg/m^3^ (mean, 22.7 μg/m^3^; standard deviation, 4.5 μg/m^3^) in 1987 and from 18.2 to 32.3 μg/m^3^ (mean, 23.1 μg/m^3^; standard deviation, 4.0 μg/m^3^) in 1995. A north-south gradient in the PM_2.5_ level was shown. Although concentrations showed a decreasing trend from 1998 to 2015 in most municipalities (Figure 2), those for the exposure period (1987-1995) had remained largely consistent with mean levels remaining within 20.6-24.0 μg/m^3^.

**Figure 3.**
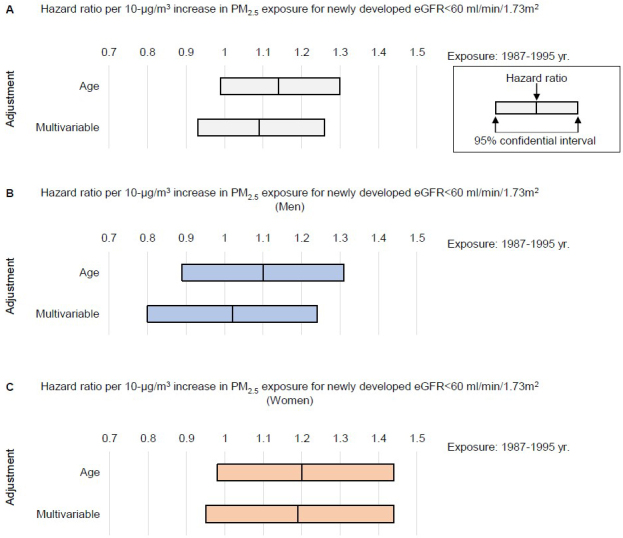
Hazard ratio of PM_2.5_ concentration for newly developed kidney dysfunction. Age-adjusted and multivariable-adjusted hazard ratio per 10-μg/m^3^ increase in PM_2.5_ exposure for incidence of eGFR of <60 ml/min/1.73 m^2^ during follow-up (A). The model was adjusted for sex, age, body mass index, blood pressure, total cholesterol, high-density lipoprotein cholesterol, glucose, treatment for diabetes, treatment for hypertension, treatment for hyperlipidemia, current smoking habit, drinking habit, and dipstick proteinuria at the baseline year of 1993. Exposure information for means of 1987-1995 was employed. HRs were also evaluated in men (B) and in women (C).

During the follow-up of 1,747,912 person-years, we observed 942 newly developed kidney dysfunction events (522 in men and 420 in women). A difference of +10 μg/m^3^ in the mean concentration of PM_2.5_ exposure around the baseline year (1987-1995) showed the age-adjusted and multivariable-adjusted HR (95%CI) for the incidence of eGFR of <60 ml/min/1.73 m^2^ during follow-up were 1.14 (95%CI, 0.99-1.30) and 1.09 (95%CI, 0.93-1.26), but this did not reach the level of statistical significance (Figure 3A). Considering sex differences, for men, the age-adjusted and multivariable-adjusted HR per +10 μg/m^3^ difference in the mean concentration of PM_2.5_ exposure for the incidence of eGFR of <60 ml/min/1.73 m^2^ were 1.10 (95%CI, 0.89-1.31) and 1.02 (95%CI, 0.80-1.24) for the 1987-1995 exposure (Figure 3B). For women, the age-adjusted and multivariable-adjusted HR were 1.20 (95%CI, 0.98-1.44) and 1.19 (95%CI, 0.95-1.44) (Figure 3C).

## Discussion

In this longitudinal prospective study of a Japanese community-dwelling population of a prefecture, we examined an association between exposure to PM_2.5_ and the onset of kidney dysfunction based on an eGFR of <60 ml/min/1.73 m^2^ during the follow-up. To the best of our knowledge, this represents the first cohort study in Japan to examine the association between air pollution, as any kind of pollutants, and the risk of incident CKD.

The current IPHS study with 77,770 participants has shown a nonsignificant association between CKD and PM_2.5_ within the range of 18.9-31.0 μg/m^3^ in 1987. Although the association did not reach statistical significance in this study, environmental exposure to elevated concentrations of PM_2.5_ may be a novel risk factor for the development of renal dysfunction, independent of classical CKD risks such as hypertension and diabetes, consistent with a recent systematic review ^(13)^ and two additional studies in the United States ^(11)^ and China ^(12)^. In the United States, a large population of 2.4 million veterans, despite a low median exposure of 11.8 (interquartile range, 10.1-13.7) μg/m^3^ across the whole country, showed that a 10-μg/m^3^ increase in PM_2.5_ concentration was associated increased risk of eGFR of <60 ml/min/1.73 m^2^ (HR, 1.21; 95%CI, 1.14-1.29) ^(11)^. In a study from China, with a high and wide range of exposures, from 31.3 to 87.5 μg/m^3^ across the whole country, an increase of 10 μg/m^3^ in PM_2.5_ was positively associated with CKD prevalence (odds ratio, 1.28; 95%CI, 1.22-1.35), from the data of about 47,000 people ^(12)^. The smaller variation of PM_2.5_ levels in this Japanese prefectural study compared to the Chinese study might be one reason why we did not detect the association. Presently, analyses of the association between CKD incidence and PM_2.5_ are limited to the country level, but not international scale, and significant differences have been found in cases with large land areas, such as the United States ^(11)^ and China ^(12)^, but not in cases with small areas, such as Denmark ^(20)^ and South Korea ^(21)^. For Japan, this cohort only has results at the prefecture level, which together suggests that the size of the region may affect the statistical significance. Therefore, it would be ideal if the epidemiology of environmental factors and CKD on a global scale were analyzed in a standardized manner, which is a challenge for the future researchers.

Moreover, we have to note the features in this study regarding temporal changes in air pollution and study length. The cohort start year for this study (1993) is the earliest among studies analyzing CKD and air pollution, which have started in the years 1996 ^(10)^, 2000-2005 ^(22), (23), (24), (25), (26), (27)^, or later. As shown in Figure 2, ambient concentrations of PM_2.5_ have clearly decreased since around 1998 due to the effects of Japanese environmental initiatives and other efforts. Although this is no doubt beneficial in terms of reducing the incidence of diseases caused by air pollution, the diminished differences in concentrations between regions make it more difficult to analyze the effects of air pollutants on disease in Japan, which may influenced nonsignificant association in this study.

The establishment of exposure periods varies widely depending on the circumstances of each study. Ideally, there should be sufficient exposure information with addresses traced from birth to subsequent observation to confirm the development of CKD. The study group relevant to this principle is determined by O’Neill et al., who analyzed a 2000-start cohort using exposure information for the last 20 years from 1982 to 2002 ^(22)^. Most other studies have used single-year results at the start of the cohort, except for the method of averaging several available years of exposure information ^(23), (28)^ and the 3 years before and after the start of the cohort ^(24), (29)^. We conducted all analyses based on available PM_2.5_ exposure information from 1987 to 1995 for all years (Figure 2), the average of the 3 years before and after (1992-1994), and a single year (data not shown) to examine CKD risk, finding no obvious differences in trends for any exposure conditions and PM_2.5_ not representing a significant risk factor for incident CKD.

To the best of our knowledge, there are no studies in Japan other than this study that have conducted annual serum creatinine measurements in a community of approximately 100,000 people for longer than 20 years. Although the primary strength of our study was the long-term follow-up that allowed investigation of the health risks of PM_2.5_, limitations to the research must also be considered. The first limitation was the lack of information on social contexts that may influence the development of CKD. For instance, other cohort studies have analyzed social factors, including income and marital status, education, and occupation as covariates ^(24), (25), (26), (27), (29)^, and such social factors could be critical for adjusting residual confounders. As the second limitation, given that many of the previously reported studies of air pollution and CKD were mostly carried out over a wide area at the national level, our study is limited to a single prefecture in Japan, which raises issues of sensitivity and difficulties in generalizing to the entire country. The third limitation was the precision of exposure information leading to potential exposure misclassification. Other studies have used postal codes to identify locations and track longitudinally whether participants moved ^(22), (25)^. Our study only included the baseline municipality of residence, making it difficult to conduct an analysis that considers differences in PM_2.5_ within the municipality and changes in exposure environment due to moving. Because the subject is at the municipal level and the air pollution is at the 1 × 1-km level of spatial resolution, the differentials are possibly out of alignment with the actual exposure. These problems with traceability and precision of residential information may be the reasons for the lack of significant differences in this study. In future studies of the relationship between air pollution and health, it is desirable to obtain more precise information on residential areas, ensuring to manage study design and protection of personal information. The fourth limitation is that we also do not have information and biomaterial (serum and urine, etc.) on estimating potential mechanisms such as oxidative stress, endothelial dysfunction, and immune-related inflammation, which lead to an increased long-term risk of CKD.

In conclusion, PM_2.5_ did not represent a significant risk factor for incident CKD in this Japanese population. In the exposure range to Japanese populations, the association between PM_2.5_ and the development of CKD was not necessarily overt. Air pollution in Japan is expected to improve more and health hazards will become more difficult to detect. The study also suggested the availability of social factor information and the traceability and precision of residential information may help investigation of the relationship between environmental pollution and renal health hazards.

## Article Information

### Conflicts of Interest

None

### Sources of Funding

This work was supported by Japan Society for the Promotion of Science (JSPS) grant no. 23K11528 and the Ibaraki Prefectural Government and Grants-in-Aid from the Ministry of Health, Labour, and Welfare, Health and Labour Sciences Research Grants, Japan (Research on Health Services: H17-Kenkou-007; Comprehensive Research on Cardiovascular and Life-Style Related Diseases: H18-Junkankitou[Seishuu]-Ippan-012; Comprehensive Research on Cardiovascular and Life-Style Related Diseases: H20-Junkankitou[Seishuu]-Ippan-013; Intractable Diseases Conquest Research: H21-Nanchi-Ippan-059; Comprehensive Research on Cardiovascular and Life-Style Related Diseases: H23-Junkankitou[Seishuu]-Ippan-005; and Comprehensive Research on Cardiovascular and Life-Style Related Diseases: H26-Junkankitou [Seisaku]-Ippan-001; H29-Junkankitou-Ippan-003, 20FA1002 and 23FA1006). The corresponding author is employed by the University of Tsukuba and Hitachi Ltd., but neither funder played any additional role in data collection or analysis, the decision to publish, or the preparation of the manuscript.

### Author Contributions

Conceptualization, Investigation, and Writing - Original Draft Preparation: Kei Nagai

Supervision and Writing―Review & Editing: Shin Araki, Toshimi Sairenchi, Kayo Ueda, Kazumasa Yamagishi, Masayuki Shima, Kouhei Yamamoto, Hiroyasu Iso, Fujiko Irie

### ORCID iD

Kei Nagai: 0000-0001-5050-5291

### Approval by Institutional Review Board (IRB)

The Ibaraki Prefectural Health Study (IPHS) protocol was approved by the ethics committees of Ibaraki Prefecture (approval no. R3-4) and the University of Tsukuba (#1628-1).

### Informed Consent

Informed consent to conduct an epidemiological study was obtained from community representatives. Individual consent was not required, since the study analysis involved the secondary use of data obtained for public health practice on cardiovascular disease prevention in the local community at that time. Adhering to relevant guidelines and regulations afterward, participants were retrospectively allowed to withdraw their data from the analysis, and consent was considered to have been obtained if the participant did not decline to participate in this study.

### Data Availability

The datasets analyzed during the current study are not publicly available due to study protocol and strict privacy protection.
